# Sodium Glucose Cotransporter-2 Inhibitor Empagliflozin Increases Antioxidative Capacity and Improves Renal Function in Diabetic Rats

**DOI:** 10.3390/jcm12113815

**Published:** 2023-06-01

**Authors:** Habib Yaribeygi, Mohammad Amin Hemmati, Fatemeh Nasimi, Mina Maleki, Tannaz Jamialahmadi, Ivan Reiner, Željko Reiner, Amirhossein Sahebkar

**Affiliations:** 1 Research Center of Physiology, Semnan University of Medical Sciences, Semnan, Iran; 2 Student Research Committee, Semnan University of Medical Sciences, Semnan, Iran; 3 Urology and Nephrology Research Center, Shahid Beheshti University of Medical Sciences, Tehran, Iran; 4 Applied Biomedical Research Center, Mashhad University of Medical Sciences, Mashhad, Iran; 5 School of Nursing, Catholic University of Croatia, 10000 Zagreb, Croatia; 6 Department of Internal Medicine, University Hospital Center Zagreb, Kišpatićeva 12, 10000 Zagreb, Croatia; 7 Polish Mother’s Memorial Hospital Research Institute, 93-338 Lodz, Poland; 8 Biotechnology Research Center, Pharmaceutical Technology Institute, Mashhad University of Medical Sciences, Mashhad, Iran; 9 Department of Biotechnology, School of Pharmacy, Mashhad University of Medical Sciences, Mashhad, Iran

**Keywords:** diabetes mellitus, diabetic nephropathy, oxidative stress, SGLT2 inhibitor, empagliflozin, malondialdehyde

## Abstract

Introduction: There are several pathologic mechanisms involved in diabetic nephropathy, but the role of oxidative stress seems to be one of the most important. Sodium-glucose cotransporter 2 (SGLT2) inhibitors are a relatively new class of antidiabetic drugs that might also have some other effects in addition to lowering glucose. The aim of this study was to evaluate the possible effects of the SGLT2 inhibitor empagliflozin on oxidative stress and renal function in diabetes. Methods: Male Wistar rats were randomly divided into four groups: control, control-treated, diabetic, and diabetic-treated (*n* = 8 per group). Diabetes was induced by a single intraperitoneal dose of streptozotocin (50 mg/kg). The treated animals received empagliflozin for 5 weeks (20 mg/kg/day/po). All groups were sacrificed on the 36th day, and blood and tissue samples were collected. Serum levels of urea, uric acid, creatinine, and glucose levels were determined. The level of malondialdehyde (MDA) and glutathione (GLT), as well as the activity of catalase (CAT) and superoxide dismutase (SOD), was measured in all groups. Data were analyzed using one-way Anova and paired T-tests, and *p* ≤ 0.05 was considered significant. Results: Diabetes significantly increased urea (*p* < 0.001), uric acid (*p* < 0.001), and creatinine (*p* < 0.001) in the serum, while the activities of CAT (*p* < 0.001) and SOD (*p* < 0.001) were reduced. GLT was also reduced (*p* < 0.001), and MDA was increased (*p* < 0.001) in non-treated animals. Treatment with empagliflozin improved renal function, as shown by a reduction in the serum levels of urea (*p* = 0.03), uric acid (*p* = 0.03), and creatinine (*p* < 0.001). Empagliflozin also increased the antioxidant capacity by increasing CAT (*p* = 0.035) and SOD (*p* = 0.02) activities and GLT content (*p* = 0.01) and reduced oxidative damage by lowering MDA (*p* < 0.001). Conclusions: It seems that uncontrolled diabetes induces renal insufficiency by decreasing antioxidant defense mechanisms and inducing oxidative stress. Empagliflozin might have additional benefits in addition to lowering glucose—-reversing these processes, improving antioxidative capacity, and improving renal function.

## 1. Introduction

The prevalence of diabetes mellitus (DM) is growing worldwide [1]. This chronic disease has negative effects on different metabolic pathways and induces pathophysiologic processes involved in diabetic complications [2]. Diabetic nephropathy, that develops in many patients with diabetes, is a frequent complication of this disease [3]. It is considered the leading cause of end-stage renal disease (ESRD) and is the second cause of mortality in patients with diabetes [4]. Therefore, preventing or reducing its progression and improving renal function is one of the major goals in the treatment of patients with diabetes [4]. Although its exact pathophysiology is so far unclear, the role of oxidative damage seems to be the most important [2,5]. Oxidative damage can be associated with endothelial dysfunction in patients with diabetes, and endothelial dysfunction could be measured through the flow-mediated dilation method [6,7]. Oxidative stress is a pathologic process which develops as a result of an imbalance between reactive oxygen species (ROS) and intrinsic antioxidant defense (ADS) and can be directed toward ROS [8]. ROS are short-living but highly active biomolecules with important physiologic roles in the control of gene expression, synaptic plasticity, cell growth, memory formation, transcription factor activation, etc. [9]. They are produced as normal by-products of metabolic pathways, and because they have unpaired electron(s), they are able to bind to biological targets such as DNA, proteins, and lipids [9]. In a normal milieu, antioxidative defense systems neutralize an excess of ROS and prevent subsequent oxidative injuries [9]. However, in the diabetic milieu, they are hyper-produced and often overwhelm ADS, and induce extensive oxidative damage in biomolecules [9]. Glucose metabolism disorders such as pre-diabetes and DM are important elements in the pathogenesis of insulin resistance which is itself a potent inducer of oxidative stress [10]. It has been shown that diabetes-induced oxidative stress can be closely associated with diabetic nephropathy and that induces it through several pathophysiologic pathways [5]. Therefore, the antioxidative agents may reverse these processes and could prevent or delay renal failure development in the diabetic milieu [11].

Sodium-glucose cotransporter 2 (SGLT2) inhibitors are a relatively new class of antidiabetic drugs that effectively reduce blood glucose levels [8]. They have inhibitory effects on urinary glucose reabsorption by blocking SGLT2s activity in renal proximal tubules and, therefore, induce massive urinary glucose excretion [8]. In vitro experiments (which are not influenced by changes in the systemic milieu) with one of these drugs—empagliflozin—also suggest that SGLT2 inhibitors may directly attenuate inflammation and oxidative stress, determining a reduction in the superoxide production and enhanced expression of glutathione s-reductase and catalase in the leukocytes of diabetic patients and downregulating IKK/NFκB, MKK7/JNK and JAK2/STST1 signaling pathways in LPS-stimulated macrophages, which could represent another pathway involved in this process [12]. Recent evidence has suggested that SGLT2 inhibitors might have additional benefits due to their antioxidative effects and that they could reduce oxidative damage in the kidneys [13,14,15,16,17,18,19]. Some studies have suggested that treatment with SGLT2 inhibitors could provide dual benefits: glucose lowering and nephroprotective effects [13]. However, there is not enough evidence to confirm their benefits from the kidneys. Therefore, the aim of this study was to evaluate the possible antioxidative effects of SGLT2 inhibitors on the kidneys and to assess their antioxidant effects on the markers of renal function in the diabetic milieu, thus providing more evidence to support the renal benefits of SGLT2 inhibitors. 

## 2. Methods

### 2.1. Animals

Male healthy Wistar rats (200–220 g) were purchased from the animal house of the physiology research center in Semnan, Iran. They were kept in standard polyester cages (three rats per cage) in a laboratory room under a standard temperature (22 ± 2 °C) and humidity (55 ± 5%) with 12 h of light and dark cycles. They had free access to water and standard rodents’ chow throughout the study except before measuring the glucose levels. They were divided randomly into four groups: control (C), control + empagliflozin (CE), diabetes (D), and diabetes + empagliflozin (DE) (*n* = 8 per each group). 

### 2.2. Diabetes Induction and Treatment 

**Figure 1:**
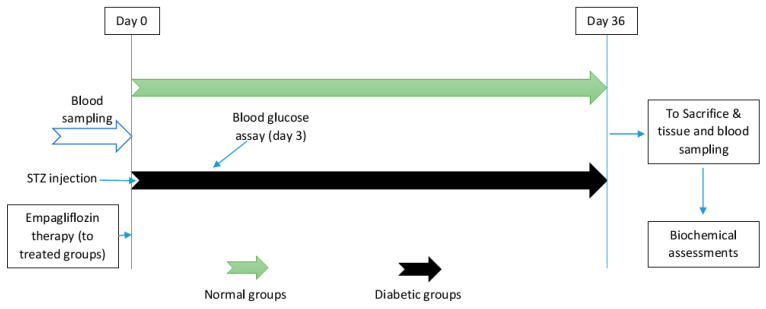
Timeline of the current study. Final biochemical assays were performed on the collected blood samples and kidney tissues. STZ: streptozotocin.

Diabetes was induced by an intraperitoneal injection of streptozotocin (STZ) (Sigma Aldrich, St. Louis, MO, USA) (50 mg/kg). After 72 h, blood samples were collected from the rat’s tail vein for a blood glucose assessment using a standard glucometer (Easy Gluco, Anyang-si, Republic of Korea), and rats with blood glucose above 220 mg/dL were considered diabetic before being randomly divided into two diabetic groups. Empagliflozin was dissolved daily in a suspension (5%) of CMC (carboxy methyl cellulose) and then was prescribed daily (20 mg/kg) by intragastric gavage with the two treated groups (CE and DE) for 5 weeks (Figure 1). The dose and duration of empagliflozin therapy were determined based on previous studies [20,21].

### 2.3. Blood and Tissue Sampling 

Blood samples were collected from the animals twice. At day zero, it was collected from the rats’ tail veins after 10 h of overnight fasting by inducing deep anesthesia through an intraperitoneal injection of ketamine (80 mg/kg) and xylazine (10 mg/kg). At the end of the 5th week, and after 10 h of overnight fasting, all rats were anesthetized, again by drugs, and were sacrificed by carbon dioxide. Then, blood (directly from the heart) and kidney tissue samples were collected immediately. Blood serum was separated immediately by centrifugation (3000 rpm for 10 min), and the samples were kept at −20 °C for biochemical tests, including glucose, creatinine, urea, and uric acid concentration measurements, which were all performed using standard commercial kits. The removed kidneys were kept at −20 °C until oxidative stress indicators such as the malondialdehyde (MDA) and glutathione (GLT) contents could be assessed, as well as the activities of the enzyme catalase (CAT) and superoxide dismutase (SOD). 

### 2.4. Tissue Preparation

The collected renal tissues were weighed and homogenized on ice by a specialized electric tool after adding a homogenization medium (phosphate buffer (0.1 mol, pH = 7.4)). Homogenized mixtures were then centrifuged (at 4 °C and 4000 rpm for 20 min), and the supernatants (as the cytosolic extract of the renal tissues) were removed and stored at −80 °C for oxidative stress indicators. The following indicators were measured. 

#### 2.4.1. Superoxide Dismutase (SOD) Activity 

SOD is the main element of the enzymatic antioxidant defense system. The activity of this enzyme can be assessed through the method of Winterbourn et al., which is based on SOD’s ability to inhibit the reduction in nitro-blue tetrazolium by superoxide anion [22]. This method was performed as follows: a 0.067 mole of potassium phosphate buffer (pH = 7.8) was added to 0.1 moles of EDTA (ethylenediaminetetraacetic acid) containing 0.3 mM sodium cyanide, 1.5 mM nitro-blue tetrazolium and 0.1 mL of the sample. Then, 0.12 mM riboflavin was added to each sample to activate the reaction and was incubated for 10 min. The sample optical absorbance was recorded at 560 nm for 5 min on a spectrophotometer. The amount of enzyme required to produce a 50% inhibition was taken as 1 Unit (U), and the results were expressed as U/mL.

#### 2.4.2. Catalase (CAT) Activity

CAT is another enzyme that is involved in cellular antioxidant defense. The activity of this enzyme was calculated using the method of Abebi [23]. A mixture containing 0.85 mL of the potassium phosphate buffer 50 mM, pH 7.0, and 0.1 mL of the homogenate was incubated for 10 min at room temperature. The reaction was triggered by the addition of 0.05 mL H_2_O_2_ (30 mM prepared in potassium phosphate buffer 50 mM, pH = 7.0). Then, the optic absorbance reduction was recorded by a spectrophotometer for 3 min at 240 nm spectrum. Enzyme activity was expressed as U/mL (1 µmole H_2_O_2_).

#### 2.4.3. Glutathione (GLT) Content

The GLT content was measured through the method of Tietze [24]. The cellular protein content of the collected supernatant was precipitated by the addition of sulfosalicylic acid (5%), which was added and removed by centrifugation (4000× *g* for 15 min). Then, the GLT content was assessed as follows: 100 µL of the protein-free supernatant was added to 810 µL of 0.3 mM Na_2_HPO_4_ and 90 µL of DTNB (5,5′-dithiobis(2-nitrobenzoic acid) in 0.1% sodium citrate. The DTNB absorbance was recorded at 412 nm for 5 min. A standard curve for GLT was recorded, and the sensitivity of the measurement was determined between 1 and 100 µM. The level of the GLT was estimated in µMol/mL.

#### 2.4.4. Malondialdehyde (MDA) Content

MDA is a toxic byproduct and marker of lipid peroxidation and oxidative damage. Its concentration was assessed through the method of Satoh et al. [25]. In total, 0.5 mL of the supernatant and 1.5 mL of 10% trichloroacetic acid were mixed by centrifugation (4000× *g* for 10 min). Then, 1.5 mL of the supernatant and 2 mL of thiobarbituric acid (0.67%) were added and placed in a hot water bath in sealed tubes for 30 min and afterward allowed to chill at room temperature. Then, 2 mL of N-butanol was added, and the mixture was centrifuged (2000× *g* for 5 min). The resulting supernatant was removed, and its optic absorbance was measured at 532 nm on a spectrophotometer. The MDA content was determined using 1,1,3,3-tetraethoxypropane as a standard. MDA’s concentration was expressed in nMol/mL. 

### 2.5. Data Analyses

The Kolmogorov–Smirnov test was used to examine the normal distribution of collected data. Then, one-way ANOVA was used to assess the possible differences among the analyzed factors between groups. A paired sample T-test was also used to assess the possible differences in the groups before and after the interventions. Tukey’s test was applied post hoc. Data were expressed as the mean ± SD (standard deviation), and *p* < 0.05 was considered to be a significant difference. 

### 2.6. Ethical Considerations

All ethical protocols regarding animal rights and complying with the demands of local and global ethics committees as well as NIH Guidelines for the care and use of experimental animals were followed throughout the study. This study was approved by the research ethics committee of Semnan University of medical sciences (IR.SEMUMS.RC.1400.263). 

## 3. Results

Table 1 shows the values of serum glucose (mg/dL) in all experimental groups at days 0 and 36 of the study. The STZ injection significantly increased it to 268 ± 22 (*p* = 0.002), causing diabetes, but empagliflozin significantly decreased it to 131 ± 11 mg/dl (Table 1).

**Figure 2:**
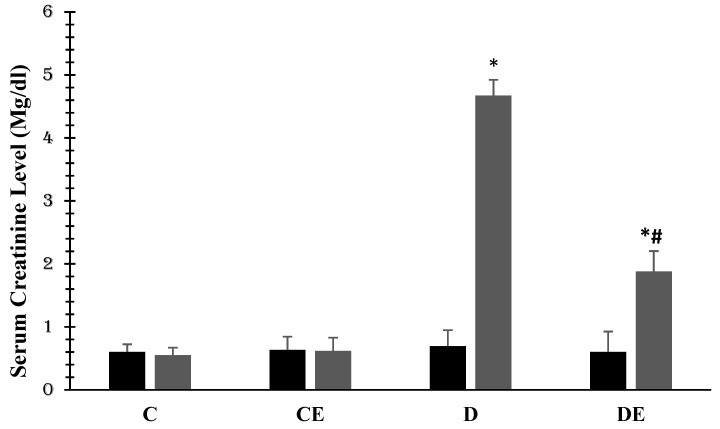
Mean values of serum creatinine (mg/dL) in all experimental groups. Diabetes induction increased this value (*p* < 0.001), but empagliflozin therapy reduced it (*p* < 0.001) at days 36th (* (*p* < 0.001)) showing a significant difference when compared with the control (C) group; **#** (*p* < 0.001) and a significant difference when compared with the diabetic (D) group (1 = day 0 or first examination, 2 = day 36). (C = control, CE = control plus empagliflozin, D = diabetes, DE = diabetes plus empagliflozin).

Figure 1 shows the changes in the mean value of serum creatinine (mg/dL) in all the groups. In the control group, this value was at the beginning and at the end 0.605 ± 0.12 and 0.55 ± 0.15, respectively. In diabetes, the value was significantly increased to 4.67 ± 0.254 (*p* < 0.001, compared with the control group). Empagliflozin had no significant effect on the blood creatinine in normal animals, but it reduced serum creatinine in diabetic animals to 1.88 ± 0.213 (*p* < 0.001, compared with the diabetic group) (Figure 2). 

**Figure 3:**
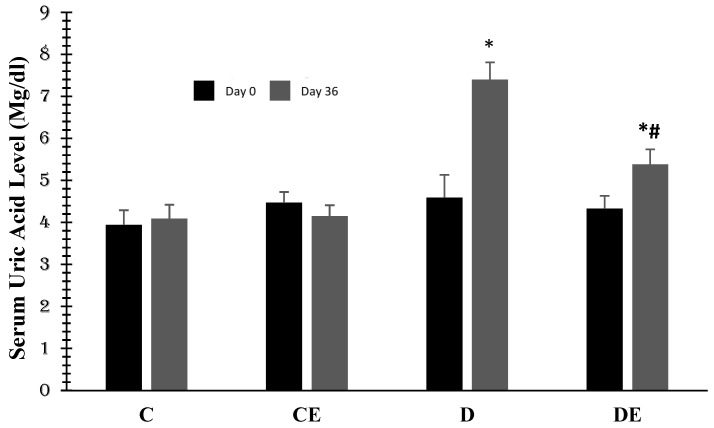
Mean values of serum uric acid (mg/dL) in all experimental groups; diabetes induction increased it (*p* < 0.001) in the D group, but empagliflozin therapy reduced it (*p* < 0.03) at days 36th. (* (*p* < 0.001)) with a significant difference when compared to the control (C); **#** (*p* = 0.03) and a significant difference when compared to the diabetic (D) group (1 = day 0 or first examination, 2 = day 36). (C = control, CE = control plus empagliflozin, D = diabetes, DE = diabetes plus empagliflozin).

Figure 3 demonstrates the changes in the mean values of serum uric acid (mg/dL) in all the groups. There were no significant differences in the control and control-treated groups. The induction of diabetes significantly increased serum uric acid to 7.4 ± 0.41 (*p* < 0.001 compared to the control group). However, empagliflozin reduced it to 5.38 ± 0.36 (*p* = 0.03) when compared with the D group of diabetic rats. 

**Figure 4:**
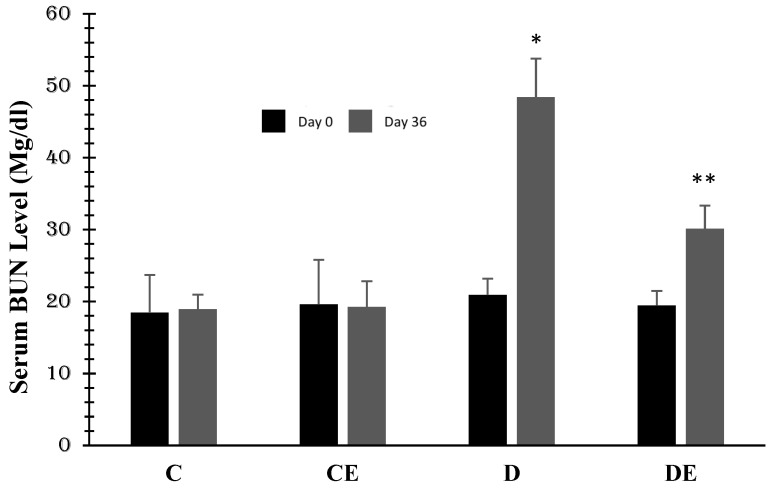
Mean values of serum BUN (mg/dL) in all groups. Diabetes induction increased it in the D group when compared with the control group (*, *p* < 0.001), but empagliflozin reduced it when compared with the D group (**, *p* = 0.03) (1 = day 0 or first examination, 2 = day 36). (C = control, CE = control plus empagliflozin, D = diabetes, DE = diabetes plus empagliflozin).

Changes in serum BUN (mg/dL) in all experimental groups are presented in Figure 3. There were no significant differences in BUN levels in the control and control-treated groups. Diabetes induction significantly increased BUN to 48.41 ± 8.21 (*p* < 0.001, compared to the control group) on the 36th day. However, empagliflozin therapy decreased it to 30.1 ± 6.59 (*p* = 0.03) when compared with the D group of diabetic rats (Figure 4). 

**Figure 5:**
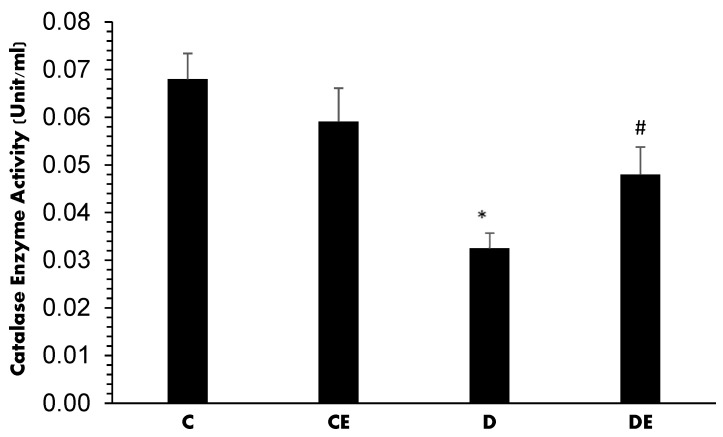
Mean CAT activity (Unit/mL) in all experimental groups. Diabetes induction decreased this parameter significantly when compared with the C group (*, *p* < 0.001)), but empagliflozin improved it when compared with the D group (**#**, *p* = 0.035). (C = control, CE = control plus empagliflozin, D = diabetes, DE = diabetes plus empagliflozin).

Figure 5 shows the CAT activity expressed as unit/mL in the kidney from all experimental groups. In normal and normal-treated animals, it was 0.068 ± 0.005 and 0.059 ± 0.007, respectively, with no significant difference. Diabetes induction significantly decreased to 0.0325 ± 0.01 (*p* = 0.01) when compared with the control (C) group. However, empagliflozin therapy increased it to 0.048 ± 0.005 (*p* = 0.01) when compared with the diabetic (D) group (Figure 5).

**Figure 6:**
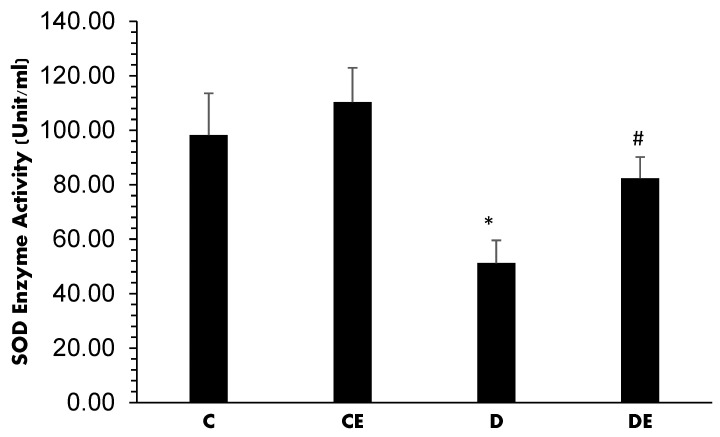
Mean values of SOD activity (Unit/mL) levels in all experimental groups. Diabetes induction decreased it when compared with the control group (*, *p* < 0.001), but empagliflozin increased it when compared with the D group (**#**, *p* = 0.02). (C = control, CE = control plus empagliflozin, D = diabetes, DE = diabetes plus empagliflozin).

The changes in SOD activity (expressed as Units/mL) are presented in Figure 5. SOD activity was 98.23 ± 15.3 and 108.25 ± 12.6 in the control and control-treated groups, respectively, with no significant difference. Diabetes decreased significantly to 51.24 ± 8.32 (*p* < 0.001). However, empagliflozin significantly increased it to 82.36 ± 7.84 (*p* = 0.02) when compared with the diabetic (D) group (Figure 6).

**Figure 7:**
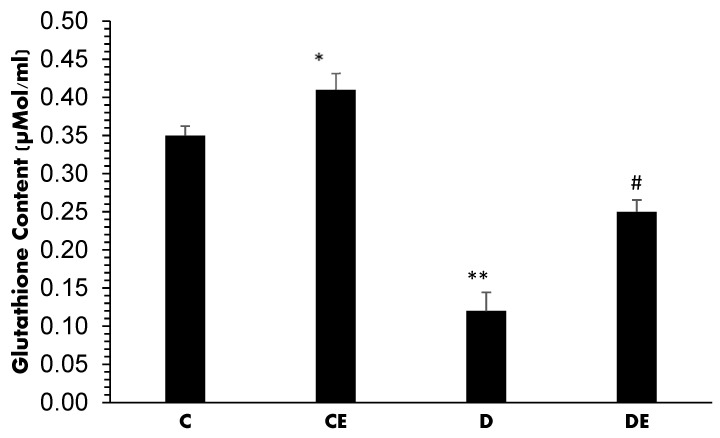
Mean values of GLT contents (µMol/mL) in all experimental groups. Diabetes induction decreased the GLT content when compared with the C group (**, *p* < 0.001). Empagliflozin improved it in non-diabetic (CE) (*, *p* = 0.045) and diabetic (DE) (**#**, *p* = 0.01) animals. (C = control, CE = control plus empagliflozin, D = diabetes, DE = diabetes plus empagliflozin).

The changes in GLT concentrations (expressed as µMol/mL) are presented in Figure 7. In normal (C) and normally treated (CE) rats, the GLT content was 0.35 ± 0.012 and 0.41 ± 0.0215, respectively, with a significant difference (*p* = 0.045). Diabetes induction significantly decreased this value to 0.12 ± 0.024 (*p* < 0.001). However, treatment with empagliflozin increased it to 0.25 ± 0.014 (*p* = 0.01); however, this was still lower compared to the control group (*p* < 0.001). 

**Figure 8:**
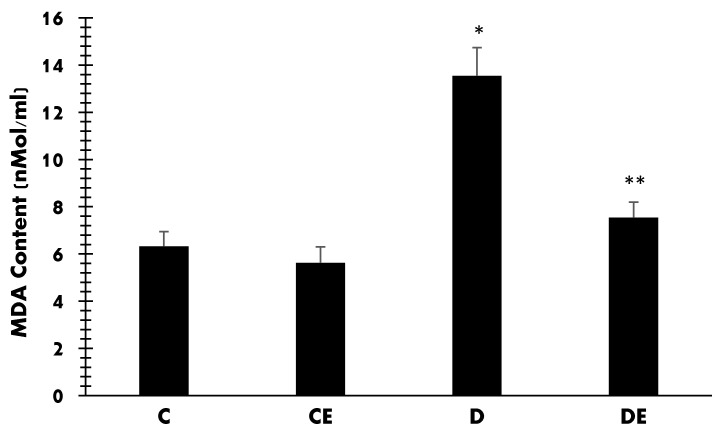
Mean values of MDA content (nMol/mL) in all groups. Diabetes induction increased it when compared with the control group (*, *p* < 0.001), but empagliflozin therapy reduced it when compared with the D group (**, *p* = 0.04). (C = control, CE = control plus empagliflozin, D = diabetes, DE = diabetes plus empagliflozin).

The MDA contents (nMol/mL) in all groups are shown in Figure 8. In control (C) and control-treated (CE) animals, the MDA contents were 6.32 ± 0.62 and 5.62 ± 0.68, respectively, with no significant difference. Diabetes increased MDA significantly to 13.54 ± 1.25 (*p* < 0.001). However, empagliflozin reduced it to 7.54 ± 0.65 (*p* < 0.001).

## 4. Discussion

This study demonstrated that the antidiabetic drug empagliflozin could have dual benefits—not only lowering glucose but also providing benefits for the kidneys in diabetes. It also showed that chronic uncontrolled hyperglycemia suppresses ADS capacity and induces oxidative stress and toxic byproduct generation leading to renal insufficiency and diabetic nephropathy. However, the SGLT2 inhibitor empagliflozin reversed these processes and improve the renal function by increasing ADS and protecting against oxidative injury in renal tissues.

Nephropathy induced by diabetes is the leading cause of ESRD and kidney failure in patients of hemodialysis [26,27] with diabetes after a median follow-up of 15 years [28,29]. This chronic complication of diabetes was primarily characterized by an increased urinary albumin excretion or decreased estimated glomerular filtration rate (eGFR), or both [29]. It is often accompanied by biochemical changes in the serum, such as increased creatinine, BUN, and uric acid levels, as well as a reduced albumin concentration [30,31]. The exact pathophysiology of this entity is so far not clear; however, the role of oxidative stress seems to be the most important [5]. As was stated before, the diabetic milieu is usually accompanied by different levels of oxidative stress due to the activation of different pathophysiologic pathways and pro-oxidant enzymes, e.g., NOX (nicotinamide adenine dinucleotide phosphate oxidase) [9]. Oxidative stress has a negative impact on podocytes, and it destroys the negative charges of the filtration barrier, allowing proteins (mostly albumin) to cross this barrier [5]. Oxidative stress also has a significant effect on the extensive vascular network of the kidneys, inducing vascular injury by activating the renin-angiotensin system and causing hemodynamic changes alongside the formation of atheromatous plaques in big vessels [5,32]. Therefore, suppressing it and reducing free radical levels by using antioxidants could be an effective adjuvant approach for the treatment of diabetic nephropathy [11]. 

In this study, the induction of diabetes was accompanied by suppressing ADS in the renal tissue. It reduced the activity of SOD, CAT, and GLT concentrations which are the major elements of ADS in the kidneys. The level of MDA as a toxic byproduct of oxidative damage was also higher in diabetic kidneys compared with non-diabetic kidneys. This finding was in accordance with the results of our previous studies [33,34,35] as well as some other reports [36,37]. Diabetes has negative impacts on ADS at several steps: at the transcriptional, post-transcriptional, and functional levels [9]. It can change the regulation and expression of enzymes such as SOD and CAT [38,39]; it can also reduce GLT synthesis [40] and increase its utilization [41], which all leads to reduced levels of antioxidants and decreased ADS which, in turn, increases the risk of oxidative stress [9]. The findings of this study showed that the diabetes-induced decrease in ADS caused oxidative stress in the kidneys [5]. These changes were accompanied by renal insufficiency, which was marked by an increase in BUN, uric acid, and creatinine levels in the serum. Treatment with the SGLT2 inhibitor empagliflozin improved diabetes-induced diabetic nephropathy in these animals to a certain extent. In addition to improving the serum glucose level, empagliflozin improved the ADS capacity and decreased oxidative injury (as shown by MDA levels) in diabetic kidneys. This was in accordance with the findings of some previous studies, which suggested that empagliflozin might increase ADS capacity [42,43]. However, since there was little evidence concerning its effects on diabetic kidneys, this study provided such evidence. Based on the current evidence, SGLT2 inhibition by empagliflozin increased ADS capacity and provided antioxidative beneficial effects in diabetic kidneys. These effects were accompanied by an improvement in renal function, as shown by the improved serum levels of BUN, uric acid, and creatinine. However, the net effect of empagliflozin in non-diabetic animals was not significant. Diabetes induction increased uric acid, and empagliflozin reduced it. This is important to mention since in the plasma, uric acid has antioxidant properties while, on the contrary, in the cytoplasm or in the atherogenic plaque, it has a pro-oxidative role promoting oxidative stress and thus contributing to the development of cardiovascular disease. [44]. The limitations of this study include a lack of assessment of the evaluated factors using PCR, Western blotting, or ELISA. Nevertheless, previous studies [45,46] have indicated these effects at molecular levels. There is also a need for additional evaluations using genetic techniques, which should be used in further studies which are already planned. However, the findings of this study have demonstrated the effects of diabetes induction and empagliflozin therapy on the examined parameters. Another limitation is that the streptozotocin model is not the best one to evaluate the development of diabetic nephropathy in type 2 diabetes. The duration of the study was short, and the changes observed in such a limited time interval might not reflect the long-term changes in type 2 diabetes. The strength of this study is that its results suggest the anti-oxidative properties of SGLT2 inhibitor empagliflozin and its benefits on the kidneys in the diabetic milieu. 

## 5. Conclusions

Uncontrolled diabetes significantly decreased ADS and induced renal insufficiency. However, treatment with empagliflozin could provide dual benefits, not only in serum glucose reduction but also with beneficial effects on the kidneys. Empagliflozin improved ADS in the kidney tissue and protected it against oxidative damage. It could improve the parameters of renal function by providing antioxidative benefits in the diabetic milieu. 

## Figures and Tables

**Table 1:** Mean values of blood glucose in mg/dL (±SD) for all experimental groups at days 0 and 36 of the study.

Groups	Serum Glucose	
	Day 0	Day 36
Control	97 ± 11	103 ± 8
Control + Empagliflozin	96 ± 12	100 ± 9
Diabetes	268 ± 22	160 ± 13
Diabetes + Empagliflozin	268 ± 22	131 ± 11

## Data Availability

Data associated with this study could be accessed from the authors on a reasonable request.
